# Supplementary figures and images for: The regulating effects and mechanism of biochar and maifanite on copper and cadmium in a polluted soil-Lolium perenne L. system
Source: PeerJ. 2021 Aug 9;9:e11921. doi: 10.7717/peerj.11921 (PMC8359803; doi:10.7717/peerj.11921)

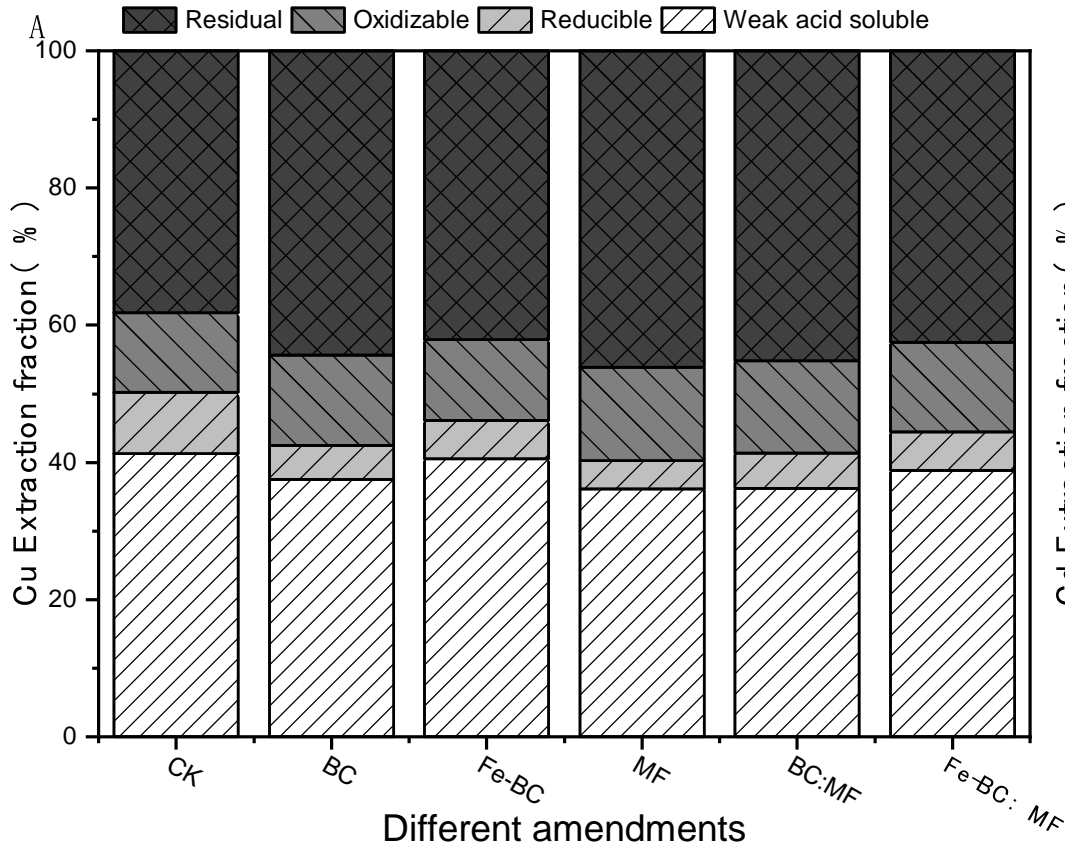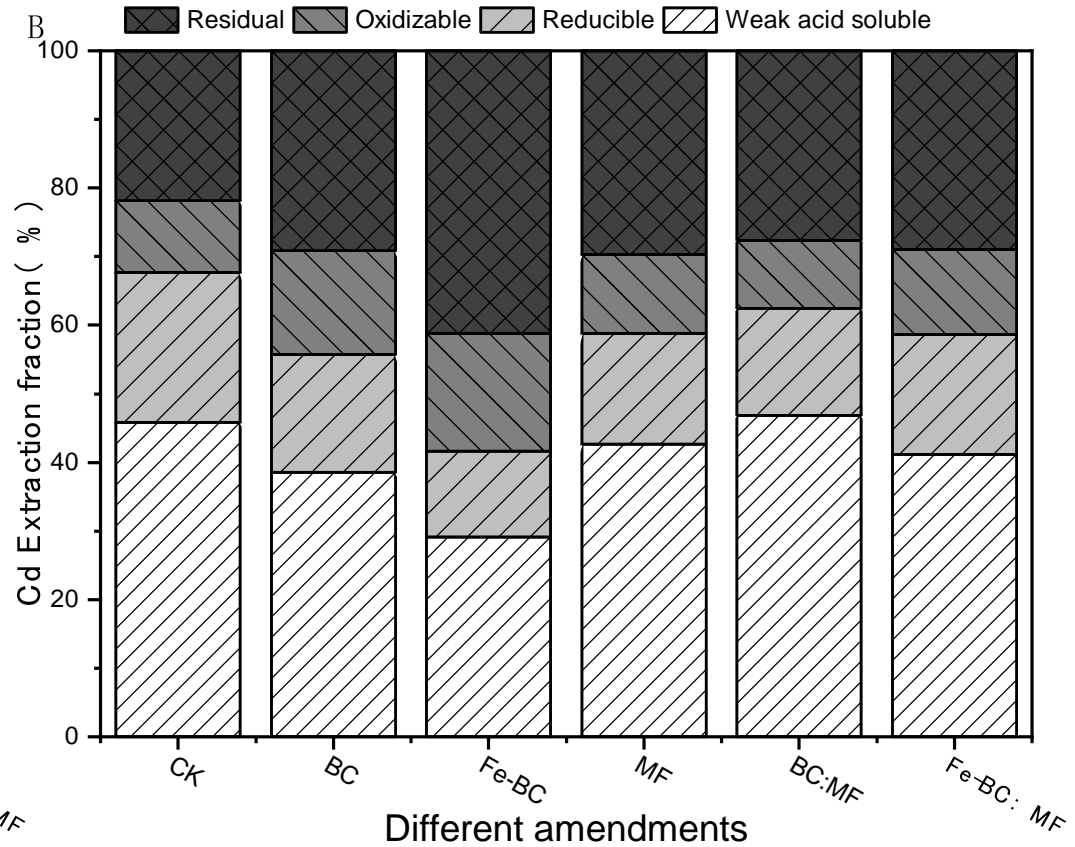

Supplement: Supplemental Information 6 — Changes of heavy metal extraction fraction percentage in soil treated with immobilizers. [file peerj-09-11921-s006.pdf]
